# Apoptosis of Adipose-Derived Stem Cells Induced by Liposomal Soybean Phosphatidylcholine Extract

**Published:** 2018

**Authors:** Reza Y. Purwoko, Iis Rosliana, Siti Sobariah, Nabila Hermana, Silvani Permatasari, Dewi Wulandari, Puji Sari, Ernie H. Purwaningsih, Chaidir, Hans-Joachim Freisleben, Jeanne A. Pawitan, Kusmarinah Bramono

**Affiliations:** 1. érpour Medical-Spa Skin and Mesotherapy Centre, Jakarta, Indonesia; 2. Department of Pharmacy, Faculty of Medicine, Universitas Indonesia, West Java, Indonesia; 3. Department of Biology, Faculty of Medicine, Universitas Indonesia, West Java, Indonesia; 4. Department of Pharmacy, Faculty of Medicine, Universitas Indonesia, West Java, Indonesia; 5. Agro Industrial Technology Development Biomedical Laboratory, Serpong, South Tangerang, Indonesia; 6. German Indonesian Medical Association, Jakarta, Indonesia; 7. Department of Histology, Faculty of Medicine, Universitas Indonesia, West Java, Indonesia; 8. Department of Dermatovenerology, Faculty of Medicine, University of Indonesia, West Java, Indonesia

**Keywords:** Adipose-derived stem cells, Apoptosis, Liposomes, Phosphatidylcholines

## Abstract

**Background:**

Recently, Phosphatidylcholine (PC) has been used as an off-label treatment for lipolysis injection, which is associated with inflammatory reaction due to sodium deoxycholate, an emulsifier, so that inflammation as side effect occurs in those patients. Liposome formulation from *soybean* lipid was thought to be a better and safer alternative. This study aimed to analyze the mechanism of Liposomal Soybean Phosphatidylcholine (LSPC) extract from Indonesian soybeans (containing 26% PC) to induce Adipose-derived Stem Cells (ASCs) death *in vitro*.

**Methods:**

Liposomes were prepared using thin film hydration method followed by a stepwise extrusion process to produce a small amount of 41.0–71.3 *nm.* Liposomal soybean phosphatidylcholine extract (LSPCE), liposomal purified PC (LPCC), and solution of PC+SD were used for comparison. Annexin V fluorescein Isothiocyanate/Propidium Iodide (FITC/PI) double staining by flow cytometry and also measurement of caspase-3 activity using ELISA were used to quantify the rate of apoptosis. ASCs viability was measured using MTT assay after induction with liposomes. Morphological changes were shown using a phase-contrast, inverted microscope and Transmission-Electron Microscope (TEM).

**Results:**

The flow cytometry results showed that cells treated with both LSPCE and LPCC showed increase in early apoptosis beginning at 6 *hr* after incubation, which was confirmed by caspase 3 measurement. MTT assay showed that both LSPCE and LPCC could decrease viability of cells. Cells treated with LSPCE and LPCC showed some rounded cells, which was an early sign of cell death. Cells treated with SD showed extensive membrane damage with necrosis features using TEM.

**Conclusion:**

The results above demonstrated that LSPCE induced apoptosis of ASCs

## Introduction

Although liposuction is highly efficient for correcting unwanted localized fat deposits, injection of lipolysis has advantages of minimal downtime, less invasiveness, and nonsurgical therapies. During the past decades, lipolysis injections using Phosphatidylcholine (PC), Sodium Deoxycholate (SD), or combination of both have been frequently used in certain countries such as United States, South America, and Europe 1–3. Phosphatidylcholine from various sources has been used as an active agent in the formulation of SD containing lipolysis injection of solutions intended to reduce subcutaneous fat 4,5, which is associated with inflammatory reaction due to the SD content 6.

PC-SD has not been approved by Food and Drug Administration (FDA) but used as a lipolysis procedure due to lack of clear mechanism that is responsible for fat loss and safety concern. SD was confirmed to induce cell death and USFDA-approved with the name Kythera® as a treatment only for adults with moderate-to-severe fat below the chin, but it was still considered off-label 7 because of SD side effects such as pain, redness and inflammation of the skin 8. SD as a detergent emulsifier causes cell and tissue necrosis and inflammation 6,9.

The effect of PC on fat cells has been studied, but its mechanism needs to be further investigated. Klein *et al* reported that Bovine Serum Albumin (BSA) solubilized PC has less effect on fat cell viability 10. A recent study confirmed the role of PC that could induce apoptosis in adipocyte cell culture 11, so PC alone might be developed into a safer lipolytic agent that causes less necrosis, and thus less inflammation and side effects. PC is not soluble in water, so it is a main obstacle to investigate PC’s efficacy. Adding SD or BSA may interfere with PC’s effect as reported in another study.

Recently, Purwoko *et al* have successfully prepared liposome that was made from Soybean PC (SPC) extract, which made the PC soluble without addition of other substances 12. This SPC extract liposome was reported in an animal study and was shown to be effective to reduce mice fat cells compared to negative control, but the mechanism needs to be further investigated. The aim of this study was to analyze the mechanism of Liposomal Soybean PC (LSPC) extract from Indonesian soybeans (containing 26% PC) to induce apoptosis of Adipose-derived Stem Cells (ASCs) *in vitro*.

## Materials and Methods

### Ethics approval

Adipose-derived stem cells were obtained from patients who underwent liposuction at érpour Medical-Spa Skin and Mesotherapy Centre, Jakarta after signing a written informed consent. This study was approved by the Ethics Committee for Medical Research, Faculty of Medicine, Universitas Indonesia (No. 255/H2.F1/ETIK/2013).

### Materials

LSPC was extracted from soybean seeds of Argomulyo variety purchased from the Indonesia Research Institute for Legumes and Tuber Crops (ILETRI), Malang, East Java Province. Purified PC of >99% purity (L-*α*-phosphatidylcholine) was purchased from Sigma-Aldrich, USA. Solutions of PC and SD (Dermastabilon® injection) and 2% SD (Deoxylise®) were imported from Aesthetic Dermal, Girona, Spain.

### Liposome preparation

Extraction and fractionation of soybean PC was done in the Agro Industrial Technology Development Biomedical Laboratory, South Tangerang, Banten Province, Indonesia. The final product consisted of 26% SPC by High Performance Liquid Chromatography (HPLC) 12. Liposome preparation was done in the Laboratory of Medical Pharmacy, Faculty of Medicine, Universitas Indonesia, Jakarta using thin film hydration (hand-shaking) method followed by a stepwise extrusion process through polycarbonate filters with 100 *nm* probes in a LiposoFast syringe. The characteristics showed that median diameter of liposomes from SPC extract (LSPCE) was 48.9 *nm* (10^th^–90^th^ percentiles, 41.0–71.3 *nm*) with polydispersity index of 0.35. They were anionic liposomes with a mean zeta potential of −17.25 *mV*. Liposomes from purified PC (LPPC) were larger with a median diameter of 68.3 *nm* (10^th^–90^th^ percentiles, 55.7–96.8 *nm*), with a polydispersity index of 0.16. The liposomes were cationic with a mean zeta potential of 58.23 *mV*. The presence and morphology of small unilamellar liposomes were further confirmed by transmission electron 12.

### Isolation, culture, and characterization of ASCs

ASCs were harvested from liposuction aspirate. Isolation and culture of the ASCs were done according to a recently published protocol 13. In brief, lipoaspirate was filtered using a fine-mesh, stainless-steel tea filter and was extensively washed in Phosphate-Buffered Saline (PBS pH=7.4) until it became clear and clean. Enzymatic breakdown was induced by adding 0.075% collagenase type I (Sigma, St. Louis, Missouri, USA). The tubes were incubated at 37°*C* for one hour and were agitated every 5 *min*. The floating yellowish lipid and remnants were discarded and the infranatant was transferred into a sterile 15 *ml* centrifuge tube and centrifuged at 1200 *rpm* for 10 *min*. Cells were re-suspended in low-glucose Dulbecco’s Modified Eagle’s Medium (DMEM) supplemented with L-glutamine, 10% Plasma-Rich Platelets (PRP), 1% amphotericin B, 1% penicillin, 1% streptomycin, and 1% heparin at 37°*C* in a humidified atmosphere containing 5% CO_2_. Cells were seeded in 12-well plates at a density of 10^5^ cells/well. Cells were observed and the culture medium was refreshed every 2–3 days. Once the cells had grown to 80% confluence, adherent cells were dissociated (TripLE^TM^ Select Enzyme, Thermo-Fisher Scientific, USA) and passaged. Cells from the third to fifth passage were collected and used for further tests.

Characterization of ASCs was done using flow cytometry (BD Stemflow^TM^ hMSC Analysis Kit, BD Sciences, USA). Cells from passage 5 were harvested, and washed with PBS and centrifuged at 1200 *rpm* for 10 *min* before analysis. The kit components included a series of cocktail antibodies, which are positive (CD73, CD90, CD105), and negative markers (CD34, CD45, CD11b or CD14, CD10 or CD79α, and HLA-DR) for Mesenchymal Stem Cells (MSCs). Based on International Society for Cellular Therapy (ISCT), MSC should express CD105, CD73 and CD90 (≥95%) and lack the expression of CD45, CD34, CD14 or CD11b, CD79α, and HLA-DR surface molecules (≤2%) 14.

### Flow cytometric (FCM) analysis of apoptosis

Cells of the third passage were seeded in a 12-well plate at a density of 150,000 cells per well. On the next day, the culture medium was replaced with media containing tested drugs: 1) liposomal SPC extract 1000 *ppm*; 2) liposomal purified PC 1000 *ppm*; 3) solution of PC+SD (Dermastabilon®) 1000 *ppm*; and 4) solution of SD 400 *ppm*. Cells were re-incubated for 2, 4, 6, and 8 *hr* at 37°*C* and 5% CO_2_.

After incubation, the culture media from each well was aspirated and collected in a conical tube of 15 *ml* which was properly labeled. Cells adherent to the base of well were dissociated using TripLE™ Select for 5 to 15 *min*. The detached cells were then aspirated and collected together into the same conical tube. Cell suspension was centrifuged at 1200 *rpm* for 10 *min*. The pellet was washed with PBS and centrifuged at 1200 *rpm* for 10 *min*. Then, it was re-suspended in 1 *ml* of Binding Buffer Solution (BBS) available in the kit (Annexin V-FITC Kit, MACS Miltenyi Biotec, USA) and was transferred to a 5 *ml*, round-bottom polystyrene tube for FCM analysis. Afterwards, Annexin V was added to the tubes. Samples were incubated for 15 *min* in the dark room. Finally, PI was added to the samples and they were incubated for at least 5 *min* before further analysis using a flow cytometer (FACS Callibur, Becton Dickinson, San Jose, CA, USA). The negative control was cells without treatment. The minimum number of event was 10,000. However, several cells did not reach this minimal requirement because many of the cells were already dead or discarded with the supernatant following the treatments. Positive Annexin V staining indicated early apoptosis, and positive PI staining indicated binding to DNA which occurred when cell membranes disintegrated.

### Microscopic evaluation

Cells with 70% confluence were incubated in culture media containing LSPCE, LPCC, commercial solution (PC+SD), and SD only. Cells without treatment served as negative control. Cells morphology was observed using phase-contrast, inverted microscopy. Transmission-Electron Microscopy (TEM) (JEOL 101, 80.0 *KV*, Tokyo, Japan) was done at Eijkman Institute, Jakarta. Samples were stained with uranyl acetate and triple lead at magnification of 3000x and 4000x.

### Measurement of caspase-3 activity

Caspase-3 activity was measured using enzyme-linked immunosorbent assay (ELISA Kit for Caspase-3, Cloud Clone, USA). The number of repeated tests in each group was doubled. The microtiter plate provided in this kit was pre-coated with an antibody specific to Caspase 3. Cells from passage-4 were seeded on a 24-well plates at a density of 50,000 cells per well. Cells were then incubated in media containing test materials (liposomal SPC extract, liposomal purified PC, Dermastabilon®, and SD) at increasing concentrations (0, 500, 10000, 500, 2000 *ppm*) for 24 *hr*. Afterwards, the cells were detached from the well and sonified 4 times for 10 *s*, and then centrifuged to obtain the cell lysate (cytoplasmic fluid) clear from debris. The supernatant and cell lysate were put into a 96-well plate coated with a specific antibody to caspace-3 and mixed with caspase-3 standard solution (0–10 *ng/ml*) for 2 *hr*. Cells were then washed with ultrapure water to eliminate antibody excess. In each well, 100 *μl* human active caspase-3 conjugate was added and was left for 1 *hr*. Cells were washed again to remove conjugate excess. A chromogenic substrate [3,3′,5,5′-tetramethylbenzidine (TMB)] was added for 30 *min*. Positive enzyme-substrate reaction produced blue color. Then the reaction was terminated by adding 100 *μl* of stopper solution containing sulphuric acid in each well. The color change was measured using a spectrophotometer at a wavelength of 450 *nm*. The concentration of Caspase 3 (CASP3) in the samples was then determined by comparing the Optical Density (OD) of the samples to the standard curve.

### MTT Assay

ASCs viability was measured using MTT assay (MTT Assay Kit, Life Technologies, USA) after treatment with test liposomes. The number of repeated tests for each group was tripled. The second-passage ASCs (at 2×10^4^ cells or 5000 cells/well) were seeded in a 96-well plate and incubated with DMEM and 10% PRP overnight at 37°*C* and 5% CO_2_. The next day, the medium was replaced by culture medium containing test liposomes (LSPCE and LPPC at increasing concentration: 0, 500, 1000, and 2000 *ppm*) for 24 *hr*. After incubation, the medium was aspirated and the cells were washed with PBS pH=7.4. Then, 100 *μl* of MTT reagent (0.5 *mg/ml*) was added to each well and incubated for 4 *hr* at room temperature in the dark. The absorbance was read at 570 *nm* using a plate reader (Bio-Rad Laboratories, California, USA).

### Statistical analysis

LPPC and LSPCE characterization was presented descriptively. MTT assay and Caspase- 3 ELISA were analyzed by independent t T test using SPSS for Windows Faculty of Medicine Indonesia University integrated version.

## Results

### ASCs isolation, culture, and characterization

**Figure 1.**
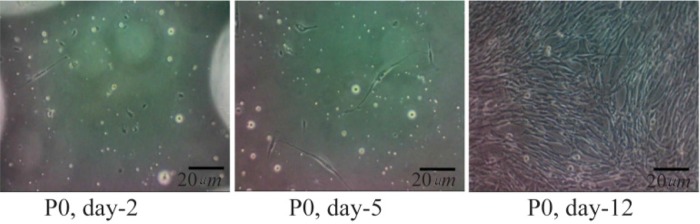
Morphology of the primary culture of adipose-derived stem cells observed by inverted microscope (magnificent 100×). P0 Day 2: ASCs were fibroblast cell like and attached in well; P0 Day 5: ASCs had been proliferated; P0 Day 12: ASCs had proliferated and confluent.

Isolations of the ASCs were achieved from six lipoaspirate donors. The cultured ASCs had fibroblast-like morphology and were adherent to the plastic culture ware from the second day. The cells underwent proliferation and were confluent between days 7 and 12 (Figure 1). In flow cytometric analysis, the ASCs showed phenotypes of MSCs as follows: CD73^+^ 99.2%; CD90^+^ 99.8%; and CD105^+^ 90.4% (see Supplement Materials). Only 1.8% of the cells expressed hematopoietic cell surface markers. Multipotency assessment of the ASCs showed that these cells were capable of differentiation into chondrocyte, osteocyte and adipocytes.

**Supplement materials:**
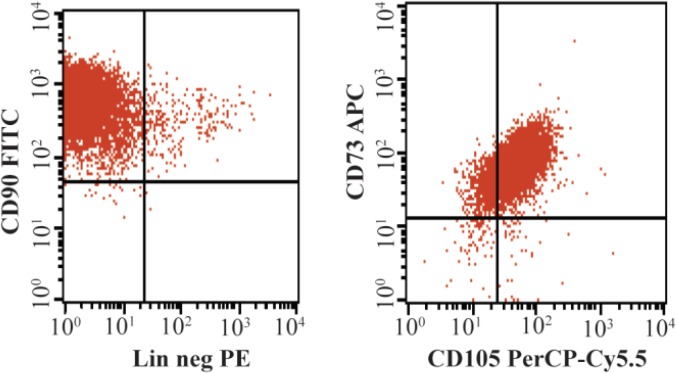
Expression of mesenchymal cell surface markers. Left: cells with positive CD90; Right: cells with positive CD73 and CD105.

### Apoptosis induced by LSPCE, LPCC, commercial PC+SD and SD

**Figure 2.**
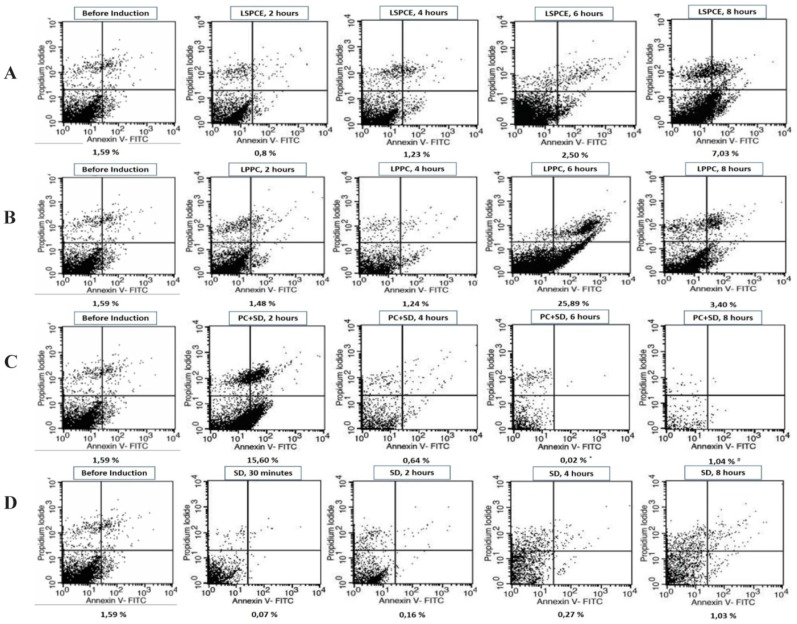
Early apoptosis assayed using Annexin V-FITC/PI by flow cytometry. The bottom of flow cytometry graphs showed percentage of early apoptosis ASCs. Annexin V-FITC at x-axis and PI at y-axis. Early apoptosis cells observed in right bottom quadrant (Annexin V+/PI -). A. Early apoptosis observed in ASCs treated with liposomal soybean phosphatidylcholine extract (LSPCE) 1000 *ppm*. B. Percentage of early apoptosis observed in ASCs treated with liposomal purified PC (LPPC) 1000 *ppm*. C. Percentage of early apoptosis observed in ASCs treated with commercial PC+SD 1000 *ppm*. After 6 and 8 *hr* induction number of cells reduced below 10,000 cells (^*^: 4890 cells and ^#^: 916 cells). D. Percentage of apoptosis observed in ASCs treated with SD 400 *ppm* (equals with SD concentration at commercial solution PC+SD 1000 *ppm*).

Flow cytometric analysis of early apoptosis and morphologies of ASCs treated with Liposomal Soy Phosphatidylcholine Extract (LSPCE), Liposomal Purified PC (LPPC), commercial solution PC+SD 1000 *ppm*, and SD 400 *ppm* can be seen in figure 2.

Flow cytometric analysis showed that the mean early apoptotic rate of negative control (cells without treatment) was 1.7%. Cells treated with LSPCE showed low apoptotic rate (lower than negative control) after the 4-*hr* induction. However, when the induction time was prolonged to 6–8 *hr*, the rate was slowly increased (Figure 2A). On the other hand, LPPC induced apoptosis after 6 *hr* of incubation. Compared with the LSPCE, the LPPC produced higher rate of early apoptosis, but then it decreased after 8 *hr* of incubation (Figure 2B).

Cells treated with commercial PC+SD showed 15.60% apoptotic rate (higher than negative control) after the 2-hour induction. It was slightly down as the induction time was prolonged 4–8 *hr* and the number of cells was substantially reduced below 10,000 cells (Figure 2C). In other case, cells were treated with SD after 30 *min* until 8 *hr* induction showed lower apoptotic rate than negative control (Figure 2D). Cells decreased with longer time induction.

**Figure 3.**
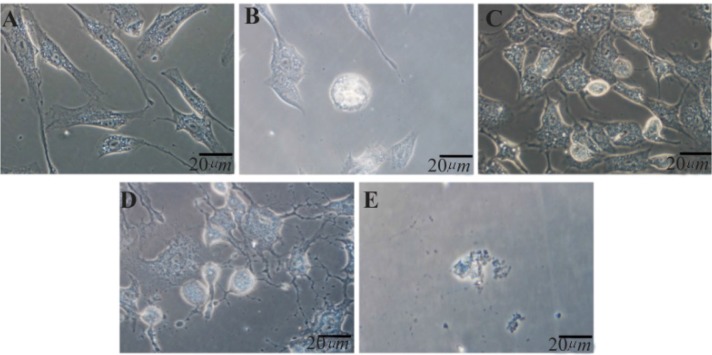
Morphological features of adipose-derived stem cells (ASCs) after 4 *hr* induction using phase-contrast, inverted microscope (magnificent 400 x). A) Control cells (without treatment) showing spindle-shape, fibroblast-like morphology; B) Cells treated with liposomal SPC extract 1000 *ppm* showing rounded cells, an early feature of apoptosis; C) Cells treated with liposomal purified PC 1000 *ppm* also showing rounded cells; D) Cells treated with PC+SD solution (Dermastabilon^TM^) showing some rounded cells, retracted cells, and cells with disrupted membrane E) Cells treated with SD only showing a damaged cell.

**Figure 4.**
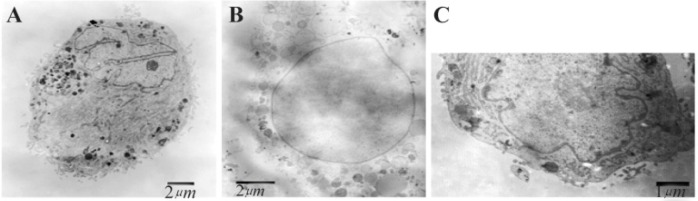
Transmission-electron microscopy of adipose-derived stem cells after induction of apoptosis. A) cells treated with liposomal SPC extract showing early sign of apoptosis with nucleus nearly fragmented B) cells treated with sodium deoxycholate showing extensive membrane damage with vesicular nucleus and disperse chromatin C) control cells without treatment showing intact membrane, normal nucleus and nucleolus.

Morphological features of adipose-derived stem cells after apoptosis induction by LSPCE, LPCC, commercial PC+SD and SD can be seen in figure 3. Control cells showed morphologies that were consistent with MSCs, *i.e*. spindle-shape similar to fibroblast (Figure 3A). Cells treated with LSPCE and LPPC showed some rounded cells, which was an early sign of cell death. Cells treated with PC+SD solution showed rounded cells, retracted cells, and disrupted membranes. On the other hand, cells treated with SD were damaged (Figure 3E). Further confirmation using TEM showed that cells treated with LSPCE showed early signs of apoptosis, *i.e*. nearly fragmented nucleus. As comparison, cells treated with SD showed extensive membrane damage consistent with necrosis features (Figure 4).

**Figure 5.**
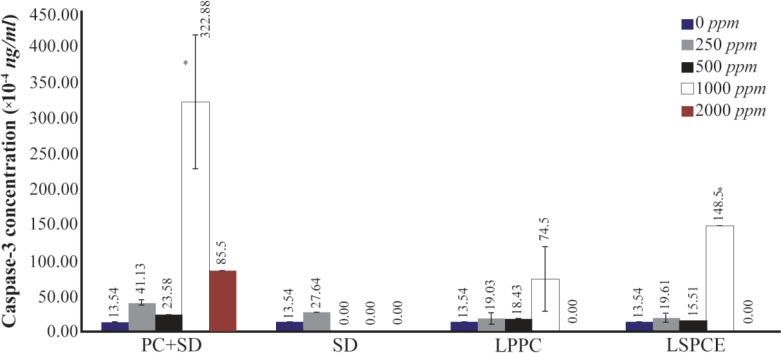
Concentration of caspace-3 *per* 1000 cells after 24 *hr* induction of apoptosis with various treatments. Cells stained by Trypan Blue. The number of repeated test in each group was double. PC+SD at 1000 *ppm* concentration significantly increase apoptosis than LPPC at 1000 *ppm* (p<0.05), statistical analysis using independent T test. (LSPCE, liposomal soybean phosphatidylcholine extract; LPPC, liposomal purified phosphatidylcholine; PC+SD, phosphatidylcholine and sodium deoxycholate (Dermastabilon®); SD, sodium deoxycholate).

Caspase-3 was measured to confirm the apoptotic mode of cell death. Relative concentration of caspase-3 was increasing as the concentration of tested formulations increased. Both liposomes from SPC extract and purified PC showed considerable caspase-3 activation at 1000 *ppm*. Highest peak was observed in cells treated with PC+SD solution (Dermastabilon®) 1000 *ppm*. No viable cells were detected after treatment with 500 and 1000 *ppm* SD. Statistical analysis showed that cells treated with 1000 *ppm* PC+SD differed significantly with negative control group (p=0.007) (Figure 5).

**Figure 6.**
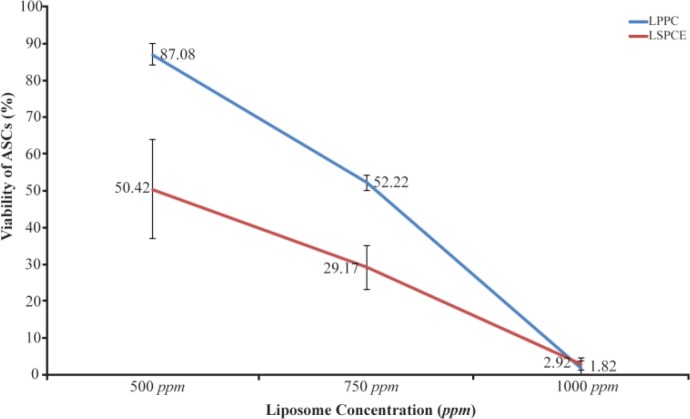
Correlation between viability of ASCs and concentration of the test liposomes. The number of repeated test in each group was triple. Statistical analysis using independent t test. (Red line showed LSPCE: liposomal SPC extract and blue line showed LPPC: liposomal purified PC).

MTT assay showed that both liposomes of SPC extract and purified PC decreased the viability of ASCs. The effect was increasing with increasing the dose of liposomes (Figure 6). The half maximal effective concentration (EC_50_) was calculated based on the graph. The effect was stronger with liposomes of SPC extract (EC_50_ =500.79 *ppm*) than the liposomes of purified PC (EC_50_ =755.26 *ppm*). Statistical analysis showed that 750 *ppm*, 1000 *ppm* and 1500 *ppm* of LSPCE group differed significantly with LPPC group with the same concentration (p<0.05, independent t test).

## Discussion

Apoptosis can be detected by many tests 15. Some of the tests did not measure cell death only, but at the same time measured common physiological (non-lethal) processes 16. Consequently, more than one method of apoptosis analysis and right timing to apoptosis test were needed. In this study, flow cytometry, ELISA, and microscopic analysis were used to detect apoptosis. Flow cytometry is one of the best techniques to detect cell death due to the possibility to use several markers and to obtain a quantitative result 17. Generally, apoptosis detection timing in cell culture (5–10 *hr*) is faster than in normal tissue (11–14 days). Timing choices to conduct an apoptosis analysis in culture depend on the type of cell, stimulant, concentration and time of induction 18.

Cells treated with LSPCE showed lower early apoptotic rate compared to negative control after 4-*hr* induction, thus showed normal variation. However, when the induction time was prolonged to 6 and 8 *hr*, the rate was slowly increased, which suggests that treatment with LSPCE caused late onset of early apoptosis.

Further, LPPC could induce apoptosis after 6 *hr* of incubation, and produced higher rate of early apoptosis compared to the LSPCE, but then apoptosis decreased after 8 *hr* of incubation (Figure 2). This fact suggested that LPPC induced much higher early apoptosis, which occurred late but massive and brief in this period of time. Apoptotic rates beyond 8 *hr* were not measured. Therefore, cells treated with LSPCE or LPPC showed increase in early apoptosis beginning at 6 *hr* after incubation. However, LPPC produced higher rate of early apoptosis compared to LSPCE. Cells treated with the commercial solution of PC+SD induced apoptosis in the first few hours, and at the same time a massive non-apoptotic cell death (probably necrosis) occurred that proved the toxicity of this formulation on the ASCs. Further, the remaining cells after two hours of incubation were far from enough to be analyzed properly. Cells treated with SD only showed apoptotic rate lower than negative control and it suggested that SD did not induce apoptosis.

LSPCE showed higher caspase-3 concentration than LPPC, suggesting that apoptosis occurs at higher rate in cells treated with LSPCE. However, FCM analyses showed that apoptosis was lower in cells treated with LSPCE. This discrepancy might be due to the fact that FCM analysis was stopped after 8 *hr*, while caspase-3 measurement was done on other cultures that were incubated overnight. It seems that apoptosis of cells treated with LSPCE continues after 8 *hr*. On the other hand, LPPC had a shorter effect and probably produced less apoptotic cells. Therefore, in this study, it was shown that liposomal formulation of soybean PC can induce apoptosis in ASCs culture.

A recent study in 3T3-L1 cells proved that PC was able to induce pre-adipose and adipose cell apoptosis. Li *et al* also found that PC in the form of liposome (LPCC) without SD induced apoptosis of MSCs 11. This fact will be used as an appropriate strategy to prevent or inhibit the occurrence of obesity.

Our study showed that LSPCE induced ASCs apoptosis at morphological obeservation with an inverted microscope and flow cytometry analysis. Compared to PC+SD or SD only, cells treated with LSPCE or LPPC showed lower rate of apoptosis. This fact means that SD is the most potent agent to induce cell death. SD content in the preparation of PC was suspected as the main active ingredient for cell death through necrosis 6. El Kamshoushy *et al* reported that injection of SD as much as 2% or 20 *mg/ml* not only caused fat cell necrosis, but also resulted in necrobiosis of the lower dermis tissue adjacent to the subcutaneous fat tissue 19. Although both LSPCE and LPPC showed lower rate of apoptosis, they are expected to be more safe. Our study showed that LSPCE was more effective in induced apoptosis than LPPC because LPPC induced much higher apoptosis, but this effect occurred late and brief in the period of time. Concentration of caspase-3 was higher in cells that were treated with LSPCE than LPPC at 1000 *ppm*. However, analysis by flow cytometry-Annexin V assay showed that reaction to LSP-CE was slower than LPPC, but the cumulative effect for 24 *hr* was higher than LPPC.

Our study showed that at a concentration of 1000 *ppm*, PC+SD worked faster and stronger than preparation of liposomes (LSPCE or LPPC). This fact might be due to the nature of PC+SD that was soluble in water and contained more PC than LSPCE. PC+SD has a possibility to work directly through Fas receptor (FasR) on cell membrane, which might be activated. Activation of the receptor can induce intracellular transduction signal to apoptosis. SD works by making holes in cell membrane and increases the permeability of mitochondrial membrane 20. Therefore, PC+SD can induce intrinsic apoptosis pathway. On the contratry, LPPC or LSPCE took longer time to internalize and trigger apoptosis.

Effectiveness of particles to induce cell death was affected by many factors, such as composition, surface charge, particle shape and size. Soya isoflavones composition in LSPCE that induced cell apoptosis had been reported by many studies and one of those studies was on adipose cell line model using 3T3-L1 cells 21,22. LSPCE has a smaller size rather than LPPC. Smaller particle size can induce cell death more effectively. Many studies on positive correlation between smaller particle size and apoptosis induction efficacy were done, such as 5 *nm* titanium dioxide particle (TiO_2_) that was shown to be more effective to induce apoptosis in mice proteoblast than 32 *nm* TiO_2_ 23. Therefore, the smaller size and existence of a synergistic effect between PC and isoflavones on LSPCE might induce cell death more effectively than LPPC.

## Conclusion

In summary, our results indicated that Indonesian soybean is a potential source for therapeutic agent. From Indonesian soybean, chemically well characterized purified Indonesian soybean extract that contains PC (PSE-PC) with sufficient level of Soybean Phosphatidylcholine (SPC) can be produced and later can be prepared as physically stable LSPCE. LSPCE can induce ASCs death through apoptotic pathway with higher therapeutic potency than PC+SD. LSPCE induced less cell necrosis than PC+SD and SD.
